# WHO Labor Care Guide as the next generation partogram: Revolutionising the quality of care during labor

**DOI:** 10.18332/ejm/138597

**Published:** 2021-07-12

**Authors:** Malitha Patabendige, Denagamage J. Wickramasooriya, Don L.W. Dasanayake

**Affiliations:** 1 Obstetrics and Gynaecology Unit, Base Hospital, Pottuvil, Sri Lanka; 2 Teaching Hospital, Karapitiya, Galle, Sri Lanka; 3 Department of Obstetrics and Gynaecology, Faculty of Medicine, University of Ruhuna, Galle, Sri Lanka

**Keywords:** WHO, Labor Care Guide, partograph, labor

**Dear Editor**,

The majority of the global burden of preventable maternal and neonatal deaths occur in low- and middle-income countries (LMICs)^1^. The World Health Organization (WHO) has recently launched a novel alternative tool, the WHO Labor Care Guide (LCG) to monitor mother and baby during labor. This is a timely need as the traditional WHO partogram failed to show any significant clinical benefit^2^. As published by Vogel et al.^3^, this LCG might possibly revolutionize labor monitoring in a woman-centered manner with shared-decision making. In their mixed-methods study, aspects pertaining to utility, acceptability, anticipated challenges and barriers have been discussed^3^. Its integration of items to promote and monitor positive birth experience is a worthwhile effort. More importantly it also covers the monitoring of the quality of care.

Similarly, WHO previously launched the Safe Childbirth Checklist which later was shown not to have a significant impact on maternal and perinatal morbidity and mortality^4^. This Safe Childbirth Checklist demonstrated improvement in essential birth practices^4,5^. A meta-analysis by Tolu et al.^6^ has reported that several aspects of maternal and neonatal morbidity and partogram utilization could be improved with this Safe Childbirth Checklist as an additional document. A question arises whether to use the Safe Childbirth Checklist and the LCG both in all settings. Since LCG is expected to replace the traditional partogram, LCG needs priority as an essential tool in all maternities worldwide, and individual centers will be able to decide the adoption of the Checklist depending on their capacity. As noted above, a large randomized controlled trial has proven that the Safe Childbirth Checklist has no effect on improving maternal and perinatal morbidity and mortality^4^.

This LCG seems to be a more robust tool that covers the first stage active phase and second stage of labor in a multifaceted way. Special attention to monitor and avoid prolonged labor, unnecessary oxytocin augmentation and caesarean deliveries, are appreciated. Promoting quality improvement as a reminder for the health staff and a tool to support audit are vital additions. Integration of new WHO recommendations on intrapartum care for a positive childbirth experience fosters its robust quality improvement aspects.

On the other hand, the starting of the active phase from 5 cm may yield better outcomes. But it leaves a fair number of women in the latent phase (<5 cm) outside of labor ward who still need some form of monitoring. In the WHO LCG manual, there is no clear updated pathway to monitor women in the latent phase, despite quoting the WHO reference in 2015^7^. Therefore, a suitable place and a method to monitor a woman with a 3–4 cm dilated cervix, who is in pain, comes as a technical necessity. Keeping the WHO partogram for the women in latent phase is also an option. Reorganization of maternity units with usual wards for non-laboring women, a bridging space/room next to the labor ward for women in the latent phase and a labor ward with space to accommodate labor companions should be the ideal set-up. Alternatively, women in the latent phase can also be included into the labor wards, as within the next few hours their labor can swiftly move into the active phase. It has been reported that updating of national policies relating to the physical layout of existing labor wards, equipment and medical supplies and standard protocols for intrapartum care, is a requirement to gain the expected benefits from this WHO LCG^3^. Arranging labor companion of choice, (‘doula’) which is a proven healthy intervention in labor support^8^, is another challenge in some settings^9^. However, it is the responsibility of the local professional bodies and policymakers to make this proven intervention work by addressing social misconceptions related to the labor companion of choice and practical ways to monitor women in the latent phase. Despite being more robust, the existing partogram should not be replaced completely and suddenly. Vogel et al.^3^ have also raised a few negative points in their qualitative findings. These include added workload, lack of a pictorial overview which leads to consuming more energy to interpret, tiny space to write, lack of staff and being reluctant to accommodate a labor companion of choice, etc.^3^ The introduction and replacement should be a gradual process with continuous audits and quality assessment. The need for training sessions, supervision, context-specific changes in each country, and practical ways of monitoring latent phase women, need to be emphasized. We should not be too quick to jump into LCG completely, and gradual implementation and pilot testing are important steps.

In summary, as a redesigned WHO partogram, the LCG is a woman-centered alternative tool to the existing partogram, and continuous future work on this tool is needed to seek any practical modifications and to ensure its expected impact.
